# Methylation level of CpG islands in GGH gene promoter in pediatric acute leukemia

**DOI:** 10.1371/journal.pone.0173472

**Published:** 2017-03-09

**Authors:** Yue Li, Sixi Liu, Huihui Wang, Huirong Mai, Xiuli Yuan, Changgang Li, Xiaowen Chen, Feiqiu Wen

**Affiliations:** 1 Division of Hematology and Oncology, Shenzhen Children’s Hospital, Shenzhen, Guangdong, China; 2 Shenzhen Institute of Pediatrics, Shenzhen Children’s Hospital, Shenzhen, Guangdong, China; Queen's University Belfast, UNITED KINGDOM

## Abstract

**Background:**

γ-Glutamyl hydrolase (GGH) regulates intracellular folates and antifolates such as methotrexate (MTX) for proper nucleotide biosynthesis and antifolate-induced cytotoxicity, respectively. In addition to genetic polymorphism and karyotypic abnormalities, methylation of CpG island 1 (CpG1) in the promoter region is found to modulate GGH activity by reducing GGH mRNA expression in acute lymphoblastic leukemia (ALL) cells. We aim to investigate methylation status of two CpG islands (CpG1 and CpG2) in the GGH promoter region in pediatric patients with ALL and acute myelogenous leukemia (AML).

**Methods:**

70B-ALL, 29 AML, 10 ITP (idiopathic thrombocytopenic purpura) and 40 healthy children are recruited in the present study. MS-HRM (methylation-sensitive high-resolution melting) and bisulfite sequencing PCR (BSP) are used to detect methylation change and its level in CpG1 and CpG2 in the GGH promoter region. GGH mRNA expression is quantified by real-time PCR. Correlation between CpG island methylation and GGH mRNA expression is assessed by statistical software.

**Results:**

Methylations of CpG1 are detected in leukemia cells samples obtained from 30.9% (21/68) of patients with ALL and 20.7% (6/29) of patients with AML. These methylations are not detected in the controls. Methylations of CpG2 are detected in leukemia cell samples obtained from 44.1% (30/68) of the ALL patients and 37.9% (11/29) of the AML patients. These percentages are significantly higher than that observed in the control cell samples: 6.0% (3/50) (Fisher's exact test, P = 0.000). The abundance of CpG1 methylation in all leukemia cell samples is classified as Grade I (methylation level is less than 10%) and the abundance of CpG2 methylation in leukemia cell samples is classified in separate grades. Our results indicate that methylation of CpG1 or hypermethylation (the methylation level is greater than 10%) of CpG2 could significantly reduce GGH mRNA expression in leukemia cells from the ALL and AML patients (ALL-CpG1: t = 4.632, P = 0.000; ALL-CpG2: t = 3.250, P = 0.006; AML-CpG1: t = -2.254, P = 0.037; AML-CpG2: t = 1.328, P = 0.202).

**Conclusion:**

Either methylation of CpG1 or hypermethylation of CpG2 in *GGH* promoter region can significantly reduce GGH mRNA expression in pediatric patients with acute leukemia, which can improve the response to treatment.

## Introduction

Antifolates such as methotrexate (MTX), acting as competitive inhibitors of folate-dependent enzymes, have been widely used for treatment of many oncological and non-oncological diseases, including acute lymphoblastic leukemia (ALL) [1]. MTX exists intracellularly as active polyglutamated MTX (MTXPG), which can stably remain in a cellular environment longer than its inactive counterpart. Due to this increased stability, it is highly effective in inhibiting the growth of cancer cells as well as normal cells [2, 3]. γ-glutamyl hydrolase (GGH) plays a central role in folate homeostasis by catalyzing hydrolysis of the active polyglutamates of natural folates and the antifolate methotrexate (MTX) into monoglutamates [4, 5]. Thus, the activity and the abundance of GGH influence the cytotoxicity of MTX by altering intracellular concentrations of MTXPG.

It has been well documented that GGH activity is significantly affected by a functional single nucleotide polymorphism (SNP) 452C→T (T127I, rs11545078) in GGH. This SNP modifies the molecular surface structure of GGH at the substrate-binding domain and decreases its binding affinity to long-chain MTXPG. It is revealed that 452C→T appears much more frequently among patients with low GGH activity than patients with high GGH activity [4, 6]. Hyperdiploid B-lineage ALL (BHD-ALL) cells with trisomy of chromosome 8 that contains a wild-type GGH allele exhibit higher GGH activity and less accumulation of MTXPG than those with disomy 8 [7]. In addition to genetic polymorphism and karyotypic abnormalities, gene expression and gene product activity are affected by epigenetic changes including DNA methylation, histone modification and miRNA editing. Cheng et al [8] reported that methylation of CpG island 1 (CpG1) in the promoter region of GGH was acute lymphoblastic leukemia-cell specific and had a significant effect on GGH expression, whereas methylation of CpG2 was common in both ALL cells and normal leukocytes but did not significantly change GGH expression. GGH activity is straight related to GGH mRNA expression in ALL cells of patients with a wild-type GGH genotype [8]. While the findings of this study are significant, we sought to build upon it as the area has not yet been investigated further. We sought to increase the scope of research, since this work includes only samples obtained from only 6 acute myelogenous leukemia (AML) patients and no normal control individuals, and no methylated CpG1 was observed in the AML cells.

The present study investigates the methylation status of the two CpG islands in the GGH promoter region in the following samples respectively: leukemia cells from pediatric patients with BNHD-ALL and AML; normal leukocytes from these patients and samples from control individuals. Our results indicate that either methylation of CpG1 or hypermethylation (greater than 10% methylation) of CpG2 could significantly reduce GGH mRNA expression in leukemia cells from ALL and AML patients. The corresponding decreased GGH abundance could lead to high accumulation of MTXPG in ALL cells, associated with better treatment response.

## Materials and methods

### Study subjects

We recruited 70B-ALL, 29 AML, 10 ITP and 40 healthy children for this study during the period of February 2014 to January 2015 at the division of hematology and oncology in Shenzhen Children’s Hospital, China. Enrolled primary ALL patients (mean age was 4.9 year) were treated with GD2008 ALL protocol, and primary AML patients were treated with SCMS AML-2009 protocol. The diagnoses of ALL and AML were based on morphologic and molecular analyses described elsewhere [9] and complete chromosomal analysis result of each patient was collected from Guangzhou KINGMED Diagnostics Ltd. Of the 70 ALL patients, 2 have trisomy of chromosome 8 in their leukemia cells. Bone marrow samples (containing more than 85% blast cells) of leukemia patients were obtained at diagnosis and normal leukocytes were isolated from peripheral blood samples obtained after the successful completion of remission induction therapy. The study was approved by the Bioethics Committee of Shenzhen Children’s Hospital and informed consent was obtained from the patients’ guardians. The authors had no access to information that could identify individual participants during or after data collection.

### Sodium bisulphite modification of DNA

Genome DNA was extracted from bone marrow samples of leukemia patients and ITP controls, as well as peripheral bloods samples from healthy control patients using FlexiGene^®^ DNA Kit (Qiagen, Hilden, Germany). The concentration of each DNA sample was measured by spectrophotometric analysis. 100ng of genomic DNA was bisulfite-converted using the Epitect^®^ Bisulfite kit (Qiagen) according to the manufacturer’s protocol. Treatment of DNA with bisulfite converts cytosine residues to uracil leaving 5-methylcytosine residues unaffected.

### Methylation analysis

Methylation primer designing software (http://www.urogene.org/cgi-bin/methprimer/methprimer.cgi) was used to predict promoter CpG islands and to design primers for amplification of bisulfite-modified DNA for methylation-sensitive high-resolution melting (MS-HRM), then bisulfite sequencing PCR (BSP) primers were used to amplify and sequence the entire CpG island to validate the HRM results (Table 1). The MS-HRM assay was first described in 2007 and is a simple closed-tube PCR-based method for easy detection of differences in DNA methylation patterns, with the superiorities of high detection rate and ability to determine the level of methylation. Amplification reactions of GGH promoter CpG1 and CpG2 contained 0.5 μl of bisulfite-treated genomic DNA, 1μl of 2.5 μM MgCl2, 0.6μl of 0.3μM primer mix, 2.9 μl ddH2O, 5 μl of 10× LightCycler^®^ 480 SybrGreen I Master (Roche, Indianopolis, USA) in a final volume of 10 μl. Amplification was performed in the LightCycler^®^480I system (Roche, Basel, Switzerland) and consisted of denaturation at 95°C for 10 min, amplification for 40 cycles at 95°C for 10s, annealing at 62°C for 15s, and final extension at 72°C for 15s. The PCR product was then subjected to HRM which included gradually heating from 40°C for 1 min to 60°C for 15s and finally the amplicon was melted at 95°C for 15s. Each sample was assessed by comparing the PCR product melting profiles between each sample and the standards with a known ratio of methylated and unmethylated templates Episcope HCT116 DKO gDNA (Takara, Dalian, China). The melting profiles of samples were compared to the melting profiles of PCR products derived from the mixes of 100, 90,75, 50, 25, 10, and 0% of fully methylated template in an unmethylated background and scored as being methylated at four levels: GradeI(0–10%), Grade II(10–25%), GradeIII (25–50%), and GradeIV (50–75%).

**Table 1:** Primer sequences of MS-HRM and BSP for two CpG islands in GGH promoter region.

Primer Name	Sequence (5′→3′)	Length
**CpG1-1f**	TTYGGTGGATAGGTTTAGTGGTGGTT	140bp
**CpG1-1r**	CACTAAAATCAAAACTAARCCAACTCCAAA	
**CpG1-2f**	GGGTATAGGTTGTTYGTATAGYGAAT	152bp
**CpG1-2r**	CCCARCCAAATTTAATAACCAARCAA	
**CpG2f**	GTTGGTTGTATTTTTAGAGTTTTTTATTGT	184bp
**CpG2r**	AAACCTATAAAAAATACAATCC	
**BSP1f**	TTTAAATTTTTTGTGAAAAAGATTGTTTAA	641bp
**BSP1r**	ACTAAACTAACCAACCCAAATCCTC	
**BSP2f**	GGATTTGGGTTGGTTAGTTTAGTTTT	760bp
**BSP2r**	AAATTCTTCATAATACACCCTCCTCC	

### Quantitative measurement

RNA were extracted using RNeasy MinElute Cleanup Kit (Qiagen). The first-strand cDNA was generated with 1μg of total RNA, random hexadeoxynucleotide primer, and RAV-2 reverse transcriptase (Takara). The cDNA samples were stored at -20°C until analysis. GGH mRNA was quantified by real-time PCR analysis using the LightCycler^®^480II system and the SYBR Green method. The primers used for amplifying GGH and β-actin were GGH-F: 5′CCAAGAAGCCCATCATCGGAA3′, GGH-R: 5′ACTGGTACAACTCTCGCACC3′; β-actin-F: 5′CGCGGCTACAGCTT- CACCAC3′, β-actin-R: 5′GGAAGCAGCCGTGGCCAT3′. The 25μl reaction mixtures were incubated in a 96-well optical plate at 95°C for 30s, followed by 40 cycles of denaturation at 95°C for 5s and annealing/extension at 60°C for 30s. Each sample was analyzed in triplicate and the expression level of GGH relative to β-actin was calculated using the basic relative quantitative analysis software.

### Statistical analysis

The obtained data with abnormal distributions were expressed as median values (5th to 95th percentiles) in tables or box plots. Data with normal distributions were expressed as mean values ($\bar{x}\pm \text{S}$). According to the different distributions of provided datas, Non-parametric models, t-test, variance analysis were used to compare expression levels between two categorical variables. The difference in distributions of methylation status between groups was performed by Chi-square test or Fisher's exact test. P values of less than 0.05 were considered statistically significant. All statistical tests were done using SPSS version 17.0.

## Results

### Methylation of CpG islands

**Fig 1:**
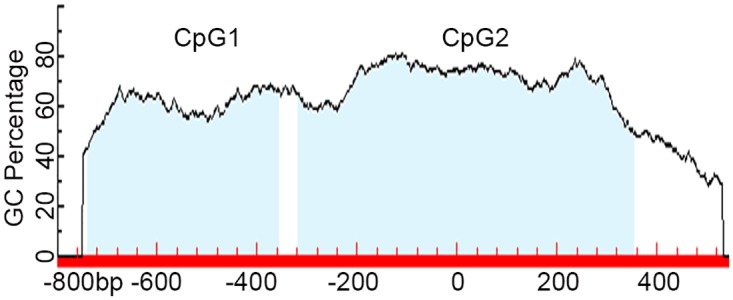
CpG islands in GGH gene. There are two CpG islands in promoter region of the GGH gene, at nt -740 to -359 (CpG1) and -319 to 355 (CpG2).

**Fig 2:**
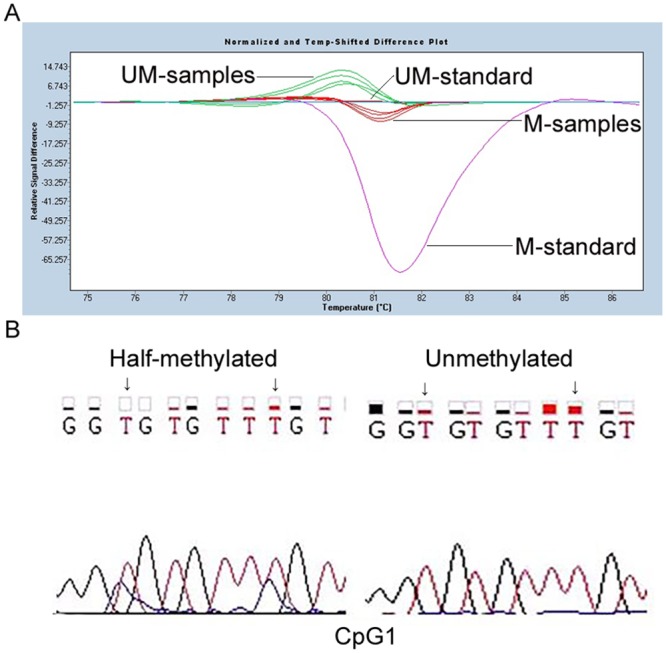
Methylation status of CpG1 in leukemia cells. A: Analysis results by HRM. B: Representative sequencing results. Methylated-standard (M-standard) and UnMethylated-standard (UM-standard) were bisulfite-converted from Methylated and UnMethylated Episcope HCT116 DKO gDNA. The different curve shapes are distinguished between methylated samples (M-samples) and unmethylated samples (UM-samples) by referring to standards.

**Fig 3:**
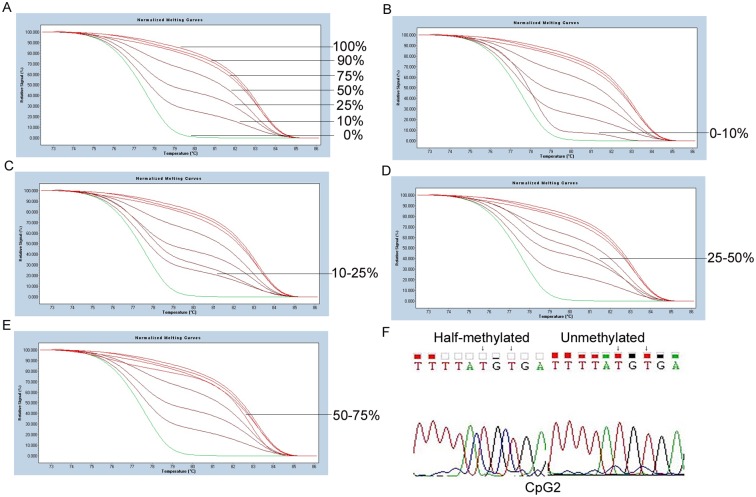
Methylation status of CpG2 in leukemia cells. A: Standard melting curve of CpG2. B, C, D and E: Represent samples of different CpG2 of GGH methylation levels: 0–10%, 10–25%, 25–50%, 50–75%. F: Sequencing results of methylated samples detected by HRM. In picture A, the seven melting profiles represent PCR products derived from the mixes of 100, 90, 75, 50, 25, 10, and 0% of fully methylated template in an unmethylated background, respectively.

Two CpG islands in the GGH promoter region at nt -740 to-359 (CpG1) and -319 to 355 (CpG2), as shown in Fig 1, were predicted using Methpimer. According to analysis by gene scanning and BSP, methylations of CpG1 were observed in leukemia cell samples obtained from 31% (21/68) of patients with nonhyperdiploid B-lineage (BNHD)-ALL and 20.7% (6/29) of patients with AML, respectively. No methylation of CpG1 was detected in the control samples. Methylations of CpG2 were detected in 44.1% (30/68) of samples obtained from ALL patients and 37.9% (11/29) of samples obtained from the AML patients, significantly higher than 6.0% (3/50), which was observed in the control samples (Fisher's exact test, P = 0.000). The representative results are shown in Figs 2 and 3, respectively. In addition, methylations of both CpG1 and CpG2 were observed in 18.0% (12/68) of the samples obtained from ALL patients and 6.9% (2/29) of the samples obtained from AML patients in the present study. Interestingly, the abundance of CpG1 methylation in all leukemia cell samples is classified as Grade I. As shown in Fig 3, the abundance of CpG2 methylation alone in the 18 ALL cell samples is classified in four separate grades: GradeI (11), GradeII (3), Grade III(2), Grade IV (2). In the 9 AML patients, the methylation of CpG2 alone is classified in three separate grades: GradeI(5), GradeII (1), GradeIII (3), Grade IV (0) cases, respectively. In the control cell samples, the CpG2 methylation is classified as Grade I in all 3 of the cases.

### Correlation between methylation and expression

**Fig 4:**
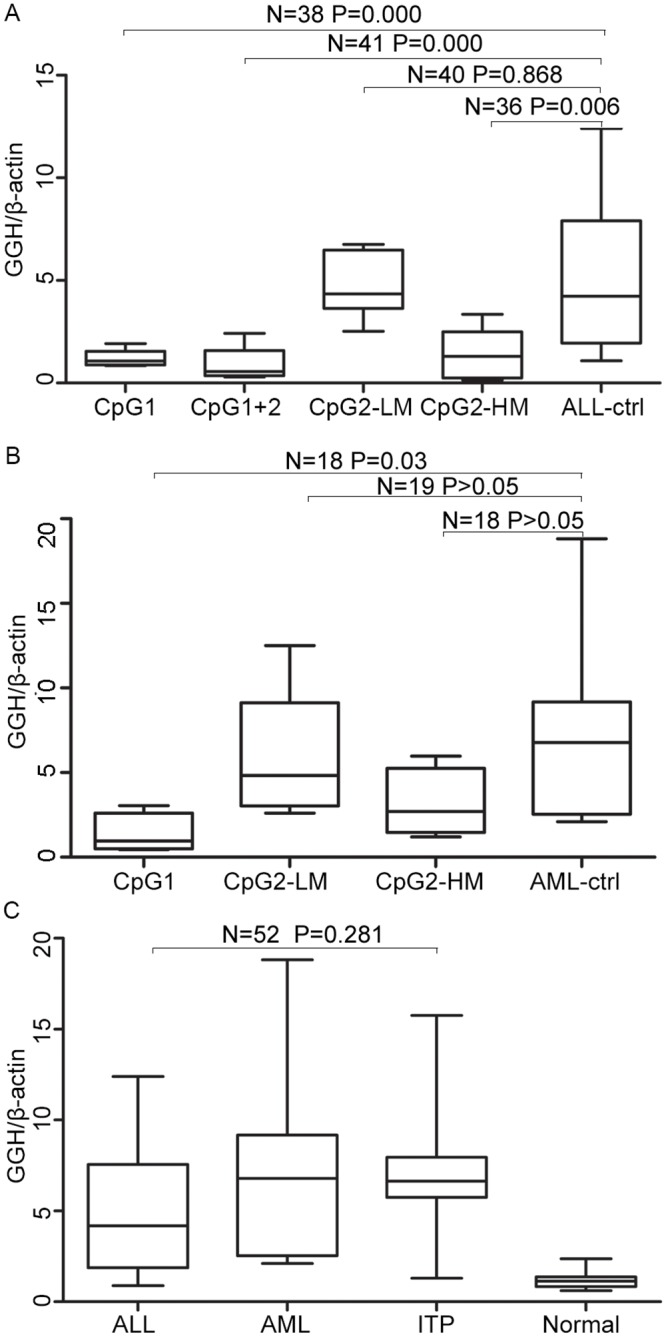
Comparison of GGH expression levels in leukemia cells. A: Samples with different methylation level in ALL group. B: Samples with different methylation level in AML group. C: Samples without CpG methylation. Differences between pairs were assessed using the exact Wilcoxon-Mann-Whitney test. Boxes represent the 5%–95% quartiles, lines in the boxes represent the median level of GGH relative expression level, whiskers represent the non-outlier range, and circles represent the outliers. LM: lower methylation level; HM: higher methylation level.

GGH mRNA abundance was quantified by real-time PCR using β-actin as a housekeeping gene to normalize expression data. Leukemia cells from 9 of 68 ALL patients exhibiting methylation in CpG1 alone show a significant reduction of GGH mRNA expression (1.0275±0.3714), compared with 29 ALL patients without methylation in CpG1 or CpG2 (4.9195±3.7293) (t = 4.868, P = 0.000). Leukemia cells with methylation in both CpG1 and CpG2 show drastically lower GGH abundance (0.8873±0.8749, t = 4.632, P = 0.000). As shown in Fig 4A, no relationship was detected between GGH mRNA expression and methylation in CpG2 alone (3.3388±2.3291, t = 1.333, P = 0.192). Further analysis shows that leukemia cells from the ALL patients whose CpG2 exhibits a methylation level greater than Grade I (which was defined as hypermethylation in this study) correlates with significantly reduced GGH mRNA expression (1.3533±1.7306, t = 3.250, P = 0.006). Simultaneously, the relationships among methylation of CpG1, methylation level of CpG2 and GGH expression in the AML patients are consistent with those in the ALL group. As shown in Fig 4B, leukemia cells from the 4 AML patients with methylation of CpG1 alone exhibit lower GGH mRNA abundance than those without methylation in CpG1 or CpG2 (t = -2.254, P = 0.037). Without statistically significant difference, but there was a tendency that GGH mRNA abundance in leukemia cells from the 4 AML patients exhibiting hypermethylation of CpG2 (3.1439±2.5028) is lower than that in the AML controls without methylation in CpG1 or CpG2 (7.1365±5.0030) and 5 AML patients with hypomethylation of CpG2 (5.8276±3.9272). In addition, without methylation in CpG1 or CpG2, GGH mRNA abundances in cells from bone marrow of ALL, AML and idiopathic thrombocytopenic purpura (ITP) control patients are significantly higher than that in peripheral blood of healthy control patients (p<0.05). No significant difference in GGH mRNA abundance is seen between leukemia and ITP cells. However, as shown in Fig 4C, GGH expression (7.1365±5.0030) is higher in primary AML cells than in ALL cells (4.9195±3.7293, t = -1.587, P = 0.121), consistent with a previous report [3].

### Comparison of GGH expression

To determine the effect of CpG1 and CpG2 methylation on GGH expression in leukemia cells and normal leukocytes, Real-time PCR was used to measure the abundance of GGH mRNA in leukemia cells and normal leukocytes (from peripheral blood when patients established complete remission (CR)) from 17 children with BNHD-ALL. The data set includes 6 samples of leukemia cells in which both CpG1 and CpG2 are methylated, one with methylated CpG1 alone and one with methylated CpG2 alone. No methylation of CpG1 was detected in the normal leukocytes. Conversely, CpG2 methylation was detected in normal leukocytes whose leukemia cells contain methylated CpG2, and the level of CpG2 methylation was not significantly different. In addition, methylation of CpG2 was detected in normal leukocytes of one sample but not in leukemia cells. In samples obtained from 7 out of 10 patients whose leukemia cells did not contain methylated CpG1, GGH mRNA was more abundant (by a factor of 1.7–10.8) in these leukemia cells than in the paired normal leukocytes. In samples obtained from the remaining 3 patients, the mRNA abundances were similar between the paired cells. In samples obtained from 3 of 6 patients whose leukemia cells were methylated in both CpG1 and CpG2, GGH mRNA abundances were lower (by a factor of 3.1, 6.3 and 10.6) in these leukemia cells than in the paired normal leukocytes. In one case, the abundance of GGH was similar between the paired cells; and in two cases, the abundances were higher in leukemia cells than in the paired normal leukocytes (by factors of 3.8 and 7.2). GGH mRNA abundances were similar between the paired cells in samples obtained from the one patient whose leukemia cells contain only methylated in CpG1 alone. These findings suggest that GGH mRNA expression could be regulated by methylation of CpG1 and other mechanisms.

## Discussion

Cancer is a disease characterized by genetic and epigenetic abnormalities. Acute lymphoblastic leukemia is the most common malignancy in children with approximately 90% long-term rates of event-free survival (EFS) in developed countries. Acute myeloblastic leukemia is less common in children and has a lower remission rate. MTX is the important component in consolidation treatment of ALL, and antileukemic effects of MTX *in vivo* are dependent on MTXPG accumulation in leukemia cells [10]. Reduced folate carrier, folylpolyglutamate synthetase, breast cancer resistance protein, binding cassette subfamily C member 1 and GGH are important for import, polyglutamation, hydrolysis and export of MTX. Many SNPs in the coding region and abnormal copies have been reported in these enzymes, all of which affect MTXPG accumulation [11–15]. Aberrated chromosome numbers such as the presence of an additional 8 chromosome, referred to as hyperdiploid, can increase GGH expression [7]. But these abnormalities cannot completely account for the differences in gene abundances and activity. Epigenetic changes such as DNA methylation are now recognized as mechanisms which regulate gene expression and ultimately contribute to the malignant phenotype [16–19]. DNA methylation is a reliable epigenetic marker that is crucial for many biological processes including cell differentiation, X-chromosome inactivation, transcription silencing, and genomic imprinting [20–23]. DNA methylation specifically occurs in CpG islands, which is predominantly in or near the promoters of mammalian genes [24].

The current study explored methylation in the GGH promoter including two CpG islands which span from the GGH promoter through the first exon and into intron 1. The results show that CpG1 methylation is specific to leukemia cells and is absent in normal leukocytes obtained from the same patients and controls. Such a finding would confirm previous reports [8]. In leukemia cells, methylation in CpG1 down-regulated GGH expression effectively and methylation in both CpG1 and CpG2 significantly reduced GGH expression. There is a significantly higher frequency of CpG2 methylation in leukemia cells compared with controls. Hypermethylation of CpG2 was found to correlate with low abundance of GGH, different from the previous report [8]. In addition, the abundance of CpG2 methylation is classified as GradeI in all three of the samples obtained from control patients. These findings suggest that not only are methylation of CpG1 and hypermethylation of CpG2 associated with GGH abundance in leukemia cells, but further that this relationship is not specific to leukemia. Considering that multiple transcriptional start sites are all in the CpG2 region, a methylation change of CpG2 could be very important in the physiological function of GGH.

MTX is an important drug in the treatment of childhood ALL, but clinical trials have shown that AML patients have low response rates to MTX, similar to the response rates of clinically resistant relapsed ALL patients. Compared with ALL cells, lower folylpolyglutamate synthase activity and higher GGH activity in AML cells might play an important role in MTX resistance [25]. In our study, methylation of CpG1 in leukemia cells from the AML patients was also detected, opposite to the previous report in which only six AML cases were studied [8]. Both the methylated CpG1 and hypermethylated CpG2 from AML cells occur less frequently than in samples obtained from ALL cells, which could contribute to a higher GGH activity in AML cells. In addition, GGH expression levels in the AML cells without methylated CpGs were higher than those in the ALL cells without methylated CpGs. It is implied that there are additional mechanisms contributing to higher GGH expression which was associated with MTX resistance in AML cells. We considered miRNA function as a possible mechanism, as we detected a reduction in miR-26b-5p expression in AML cells in another study of ours (unpublished).

Methylated CpG islands decrease GGH abundance, which could lead to high accumulation of MTXPG in ALL cells, associated with better treatment response. Therefore, when evaluating sensitivity and resistance to MTX in children with ALL, in addition to genetic polymorphism and karyotypic abnormalities, these methylation changes should be considered. In our future study, correlation between CpG island methylation and HD-MTX—related toxicity for long-term will be assessed.

In sumarry, our findings show that GGH mRNA abundance in human leukemia cells could be altered by methylation of CpG1 and hypermethylation of CpG2. Our results showed that detection of methylation level, not only methylation, could be indicative of development of cellular disease characteristics.
